# Prevalence of arbovirus antibodies in young healthy adult population in Brazil

**DOI:** 10.1186/s13071-021-04901-4

**Published:** 2021-08-14

**Authors:** Bárbara Batista Salgado, Fábio Carmona de Jesus Maués, Renato Lemos Pereira, Jannifer Oliveira Chiang, Maria Nazaré de Oliveira Freitas, Milene Silveira Ferreira, Lívia Caricio Martins, Pedro Fernando da Costa Vasconcelos, Christian Ganoza, Pritesh Lalwani

**Affiliations:** 1 Instituto Leônidas e Maria Deane (ILMD), Fiocruz Amazônia, Manaus, Amazonas Brazil; 2 Centro de Instrução de Guerra na Selva (CIGS), Manaus, Amazonas Brazil; 3 Instituto Evandro Chagas (IEC), Seção de Arbovirologia e Febres Hemorrágicas, Ananindeua, Pará Brazil; 4 Instituto de Medicina Tropical Alexander von Humboldt, Universidad Peruana Cayetano Heredia, Lima, Perú

**Keywords:** Arbovirus, Seroprevalence, Hemagglutination inhibition assay, Public health, Military personnel, Cross-reactivity

## Abstract

**Background:**

The emergence and re-emergence of infectious diseases are a cause for worldwide concern. The introduction of Zika and Chikungunya diseases in the Americas has exposed unforeseen medical and logistical challenges for public health systems. Moreover, the lack of preventive measures and vaccination against known and emerging mosquito-transmitted pathogens, and the occurrence of unanticipated clinical complications, has had an enormous social and economic impact on the affected populations. In this study, we aimed to measure the seroprevalence of endemic and emerging viral pathogens in military personnel stationed in Manaus, Amazonas state.

**Methods:**

We measured the seropositivity of antibodies against 19 endemic and emerging viruses in a healthy military personnel group using a hemagglutination inhibition assay (HIA).

**Results:**

Overall, DENV positivity was 60.4%, and 30.9% of the individuals reacted against ZIKV. Also, 46.6%, 54.7%, 51.3% and 48.7% individuals reacted against West Nile virus (WNV), Saint Louis encephalitis virus (SLEV), Ilheus virus (ILHV) and Rocio virus (ROCV), respectively. Individuals with high DENV HIA titer reacted more frequently with ZIKV or WNV compared to those with low HIA titers. Observed cross-reactivity between *Flaviviruses* varied depending on the virus serogroup. Additionally, 0.6% and 0.3% individuals were seropositive for Oropouche virus (OROV) and Catu virus (CATUV) from the family *Peribunyaviridae,* respectively. All samples were negative for Eastern Equine Encephalitis virus (EEEV), Western Equine Encephalomyelitis virus (WEEV), Mayaro virus (MAYV), Mucambo virus (MUCV) and CHIKV from the family *Togaviridae*.

**Conclusions:**

A high proportion of individuals in our high-risk population (~ 60%) lacked antibodies against major endemic and emerging viruses, which makes them susceptible for further infections. Military personnel serving in the Amazon region could serve as sentinels to strengthen global infectious disease surveillance, particularly in remote areas.

**Graphical abstract:**

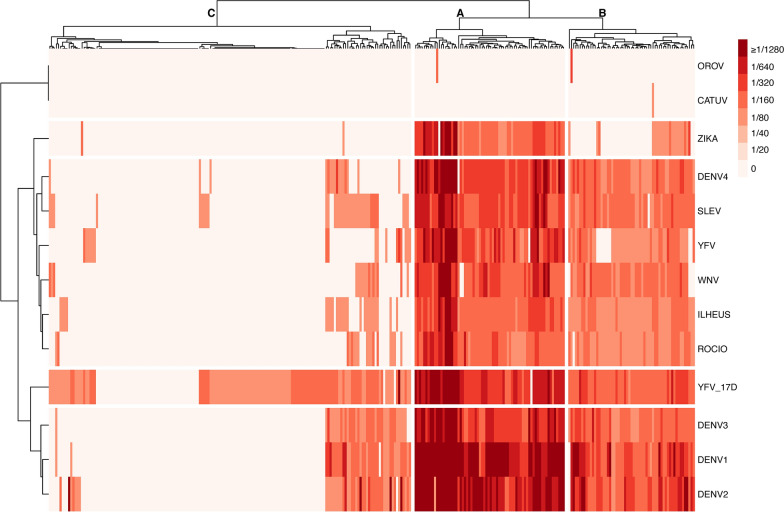

**Supplementary Information:**

The online version contains supplementary material available at 10.1186/s13071-021-04901-4.

## Background

Deforestation, urbanization and climate change have led to perpetual public health challenges of infectious diseases worldwide [1]. In the last few decades, viruses transmitted by arthropods have caused numerous outbreaks worldwide in humans and animals, causing diseases ranging from sub-clinical or mild, through febrile, encephalitic, hemorrhagic or arthritogenic disease, with a significant proportion of fatalities [2, 3].

Dengue virus (DENV), one of the foremost studied arboviruses, is still responsible for millions of infections and numerous deaths worldwide [4]. Consecutive dengue epidemics have been occurring in Brazil since 1986, and more than 4 million cases of dengue fever (DF) had already been recorded to date [5, 6]. Moreover, all four dengue virus serotypes causing human disease have been detected in Brazil, and at least two serotypes co-circulate in most of the Brazilian states; please refer to Additional file 4: Table S4 for details regarding dengue serotypes and Brazilian state [7–9]. In the absence of effective therapeutics to treat dengue disease, and low adherence to ineffective mosquito control measures, there has been an increase in dengue-associated deaths along with increasing disease incidence [5, 9].

The social and economic impacts attributed to the recent Zika virus (ZIKV) and Chikungunya virus (CHIKV) introductions, driven by the large sheer number of human infections and associated pathology in Brazil and the Americas, are well documented [10, 11]. In addition, the recent isolation of West Nile virus (WNV) from equine hosts represents an additional emerging virus circulating in Brazil [12]. It is worth pointing out that in Brazil the co-circulation of several flaviviruses (such as Dengue, Zika, Yellow fever, Saint Louis encephalitis, Ilheus and others) complicates the serological diagnosis of these emerging infections because of the extensive flavivirus cross-reactivity in the serological assays [13–16]. While many current arboviruses do not appear to cause pathology in humans or animals, this large number of widely different and highly adaptable arboviruses provides an immense resource for the emergence of new pathogens in the future [2, 11]. Epidemiological and molecular clock studies demonstrate that ZIKV and CHIKV introduction in Brazil happened up to a year before their detection; moreover, these studies also point toward clinical misdiagnosis in some cases [17, 18]. Combating these pathogens has historically been driven by the circumstances: expecting the unexpected and being prepared to respond when the unexpected occurs. Therefore, understanding the epidemiology of these emerging and endemic pathogens is necessary to ascertain their public health impact and to respond efficiently to these epidemiological and diagnostic challenges.

Furthermore, compared to civilian population, military personnel live in a communal nature, train in diverse locations like the Amazon rainforest and participate in humanitarian aid in adverse conditions, alongside suboptimal hygiene and stress in the field, which increases their risk of contracting emerging infectious diseases. Hence, soldiers can act as a sentinel population to identify emerging pathogens. However, we have few data about the serological status before or during recent arbovirus outbreaks and the role of cross-reactive antibodies against these emerging viruses. Hence, in this study we evaluated the prevalence and antibody reactivity among military personnel participating in the jungle survival course using a cell-based assay against major endemic and emerging arboviruses from three different virus families. Additionally, we performed a literature review to understand the distribution of DENV prevalence in Brazil between 1980 and 2020.

## Methods

### Sample size calculation

Seroprevalence greatly varies depending on the study population, age, sex and serological assay employed for antibody testing. Based on previous estimates of seroprevalence for arboviruses in Brazil, which ranged between < 1% to > 50% depending on the geographical location [9, 19–22], a sample size of 285 individuals was calculated using an estimated prevalence of 25% and 95% confidence interval (334,500 is the current strength of the Brazilian Armed Forces) with a desired probability of 0.05. We recruited 300 individuals (assuming a maximum of 5% of participants excluded from analyses because of missing data or analysis). Sample size calculation was performed using the Epi Info software v5.5.3 (iOS mobile).

### Study population and sample collection

The study population comprised of Brazilian army personnel participating in a jungle survival course at the Jungle Warfare Training Center (CIGS, Centro de Instrução de Guerra na Selva), located in Manaus, Amazonas State. Every year CIGS organizes up to four training camps in the Amazon rainforest, where recruits spend a maximum of 3 months inside the rainforest, training and performing exercises. We interviewed and sought ethical consent from participants before entering the rainforest and collected blood samples after the end of the jungle survival course. Number of participants varied with each training camp. Adults of both sexes ≥ 18 years were invited to participate. During the study period there were no female participants. Consecutive individuals were enrolled in this observational and cross-sectional study using convenience sampling until attaining calculated sample size of 300 between January 2014 and December 2015. A total of 4 ml of blood was drawn from each participant (using EDTA tubes, BD Vacutainer), subsequently tubes were centrifuged and plasma was separated and stored at −80 °C until further analysis.

### Hemagglutination inhibition test

The serological tests were performed at the Instituto Evandro Chagas (IEC) (Belém, Pará). Plasma samples collected were subjected to an in-house hemagglutination inhibition assay (HIA) with a titration cut-off of 1:20 plasma dilution, as previously described [20, 23, 24]. Samples were tested by HAI test to detect antibodies reactive to the following viral families: *Flaviviridae* (*Flavivirus* genus): yellow fever virus (YFV), dengue virus (DENV) serotypes 1 to 4 (DENV-1, DENV-2, DENV-3 and DENV-4), Zika virus (ZIKV), Saint Louis encephalitis virus (SLEV), West Nile virus (WNV), Ilheus virus (ILHV), Rocio virus (ROCV); *Togaviridae* (*Alphavirus* genus): Eastern equine encephalitis virus (EEEV), Western equine encephalomyelitis virus (WEEV), Mayaro virus (MAYV), Mucambo virus (MUCV), Chikungunya virus (CHIKV); *Peribunyaviridae* (*Orthobunyavirus* genus): Oropouche virus (OROV), Tacaiuma virus (TCMV) and Catu virus (CATUV).

### Spatial analysis and virus distribution

PubMed, Science Direct, LICACS, Web of Science and Medline databases were searched by keywords (Fig. 5) to identify research papers with dengue virus seroprevalence data. QGIS Software version 2.18.26 for macOS was used to plot spatial distribution of dengue prevalence in Brazilian cities; Additional file 3: Table S3 lists studies included in this analysis between 1980 and June 2020. Hot spot detection maps were plotted using publicly available data for dengue, Zika and Chikungunya virus incidences between 2014 and 2018 (Ministry of Health Brazil, https://www.saude.gov.br/boletins-epidemiologicos).

### Data analysis

To analyze the clustering of study subjects according to their HIA plasma titers against all viral species analyzed, we normalized their serological dilution values using the log_2_ of the inverse titer value, calculated with the formula normalized_titer = Log_2_[1/titer] [25]. We then constructed a heatmap plot of plasma HIA normalized titer levels with the Manhattan clustering method using the heatmap package version 1.0.12 (https://cran.r-project.org/package=pheatmap) in R for macOS with RStudio (R version 3.6.2, RStudio version 1.2.5033). The bubble plots depicting the percentages of seropositive individuals were done using Microsoft Excel 2019. Chi-square test was used to examine the differences between observed and self-reported dengue virus infection rates (GraphPad Prism version 9.1.2, Mac OS).

## Results

**Fig. 1:**
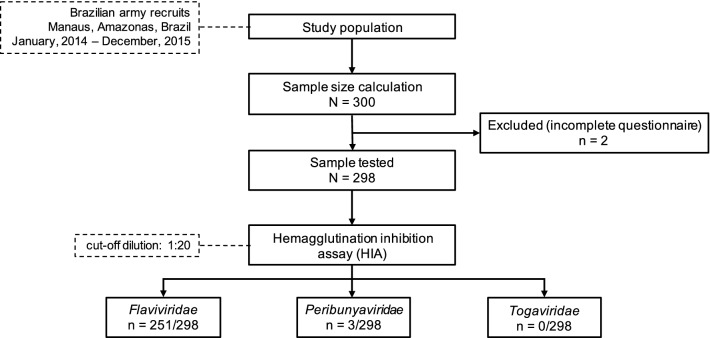
Sample collection flowchart (Strobes diagram)

**Fig. 2:**
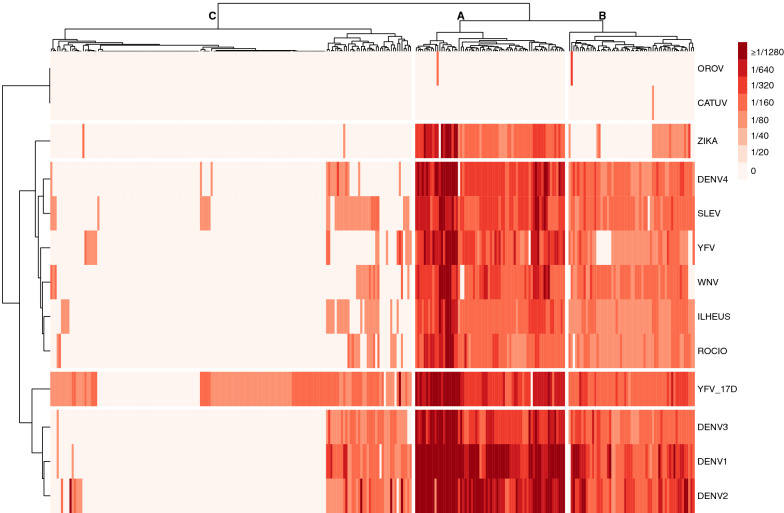
Seroreactivity against principal human flaviviruses. Patient samples (*n* = 298) were tested for antibodies against flaviviruses by hemagglutination inhibition assay (HIA). Heatmap represents the normalized antibody titers of individuals positive for at least one Flavivirus, clustered by titer. Groups: A (*n* = 70), B (*n* = 59) and C (*n* = 169)

In the current study, we performed a cross-sectional analysis to determine serological reactivity against endemic and emerging viruses. We aimed to assess the pre-Zika and Chikungunya epidemic serological status of individuals in a highly mobile group of individuals to better understand and estimate the size of the virus-exposed and susceptible populations. We recruited 300 individuals; however, two questionnaires were incomplete and removed from the analysis. Data for the 298 individuals included in the analyses are described in Fig. 1 and Additional file 1: Table S1. Serological results are summarized in Additional file 2: Table S2. All study participants were male, with a mean age of 27.98 years and median age of 26 (IQR, 24–31) years. Study participants had served in the military since attaining 18 years of age and had resided in several Brazilian states; hence, we could not ascertain the local of infection for the pathogens tested in this study. Among the three virus families tested, the majority of the individuals (251/298, 82.2%) reacted against the viruses from the *Flaviviridae* family (genus *Flavivirus*) (Figs. 1 and 2 and Additional file 2: Table S2). The prevalence for the *Peribunyaviridae* family was 0.6% and 0.3% (2/298, OROV and 1/298 CATUV, respectively). Our entire sample was negative for all five viruses tested from the *Togaviridae* family (Additional file 2: Table S2 and Additional file 5: Figure S1).

**Fig. 3:**
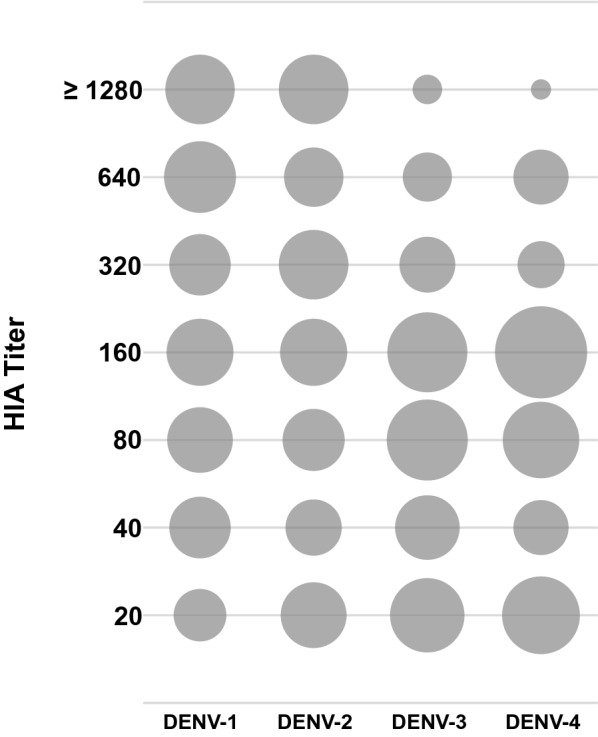
Distribution of dengue virus antibody titers. Dengue virus-positive samples were plotted on a bubble chart as function of antibody titers and virus serotype. Bubbles show the column percentages for each virus and their sizes are proportional to their values

Antibodies to DENV were predominant among soldiers and varied between 48.3% and 58.4%, depending on the serotype of the virus (Additional file 2: Table S2 and Fig. 3). We observed that during the ongoing 2014 ZIKV epidemic, and 30.9% participants had anti-ZIKV antibodies (Additional file 2: Table S2). Also 46.6% and 54.7% study participants were reactive for WNV and SLEV of the Japanese encephalitis serogroup, respectively. ILHV and ROCV, from the Ntaya virus serogroup, had a seroreactivity of 51.3% and 48.7%, respectively. Furthermore, we observed an apparent relationship between increasing age and percentage HIA positivity; however, no statistically significant differences were observed between seropositivity percentage data when stratified by age (Additional file 2: Table S2, data not shown).

We then evaluated the clustering of individuals according to their serological titers against all viruses tested. We observed that our population clustered into three groups: A (*n* = 70), B (*n* = 59) and C (*n* = 169) (Fig. 2). Group A was a distinct group where most individuals had HIA titers against all flavivirus tested. Individuals in groups A and B showed a similar clustering pattern, with high HIA titers to DENV1-3 and YFV-17D (vaccine strain), but differed in their positivity to ZIKV (98.6% vs. 35.6%, respectively), showing lower HIA titers than subjects in group A overall. Group C represented a group with 29.6% seropositivity to YF17D vaccine strain (compared to 98.6% and 100% in groups A and B, respectively) and a decreased fraction of flavivirus-seroreactive individuals overall (YFV 10% vs. 98.6 and 84.7 in groups A and B, respectively); only two individuals were seroreactive to ZIKV (1.2%). *Peribunyaviridae*-positive individuals clustered with groups A (*n* = 1) and B (*n* = 2).

**Fig. 4:**
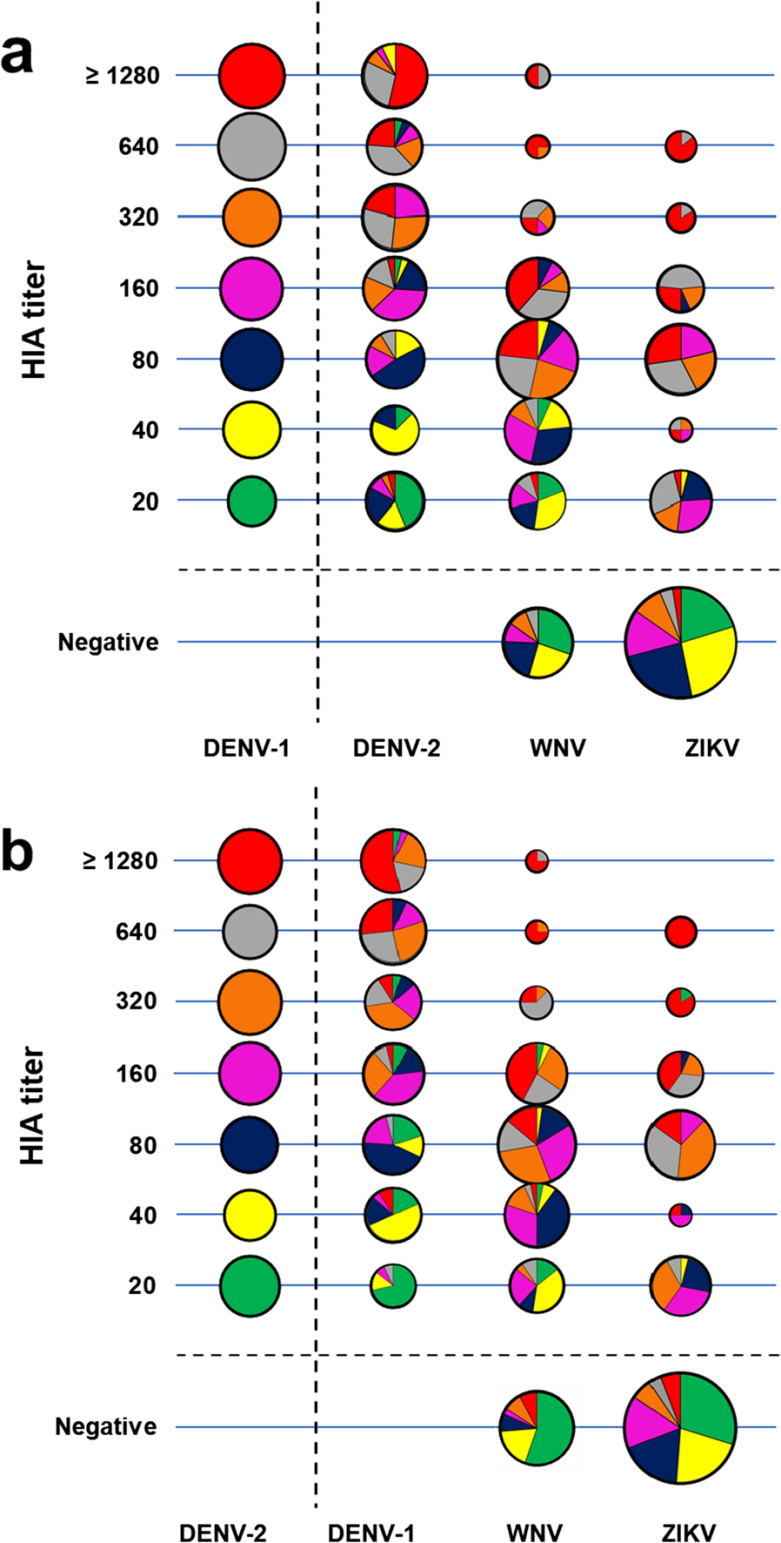
Antibody reactivity between flaviviruses. Dengue-positive samples were used to evaluate cross-reactivity between emerging viruses. DENV-1 (**a**) or DENV-2 (**b**). HIA titers were compared with DENV-2 or -1, ZIKV or WNV antibody titers. Bubbles show the column percentages for each virus, and their sizes are proportional to their values. All samples were considered positive with HIA titer ≥ 20 units. Bubbles with pie chart represent distribution of individuals with reference to DENV-1 or DENV-2 HIA reactivity

Next, we plotted a bubble chart to understand the distribution of anti-DENV antibodies per serotype (Fig. 3). A higher proportion of individuals were reactive against DENV-1 and DENV-2, ≥ 320 HIA titer. In addition, to assess the cross-reactivity between tested *Flaviviruses*, we analyzed the reactivity of DENV-1- or DENV-2-positive individuals with their corresponding reactivity for DENV-2 or DENV-1, WNV or ZIKV (Fig. 4). We observed that individuals with higher HIA titer for DENV-1 or DENV-2 reacted with WNV or ZIKV more frequently; in contrast, individuals with lower DENV titers demonstrated low or no reactivity against WNV and ZIKV. Additionally, DENV-1 or DENV-2 positive individuals reacted more frequently with WNV compared to ZIKV (Fig. 4).

Furthermore, 5% (15/295) and 17.2% (51/296) individuals self-reported previous malaria and dengue virus infection, respectively. We performed a chi-square test to assess whether self-reported dengue infection rates were similar to results observed by serological diagnosis performed in this study. We observed that serological testing showed a higher percentage of positive individuals than self-reported disease (*P* < 0.0001, data not shown).

**Fig. 5:**
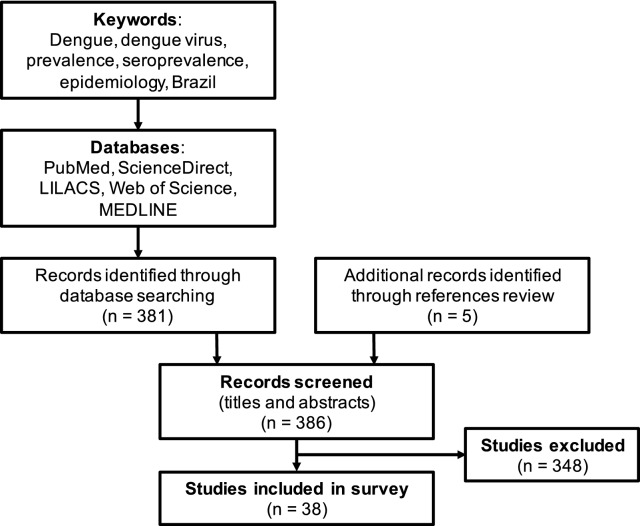
Preferred reporting items for systematic reviews and meta-analysis (PRISMA) flowchart for dengue prevalence studies

**Fig. 6:**
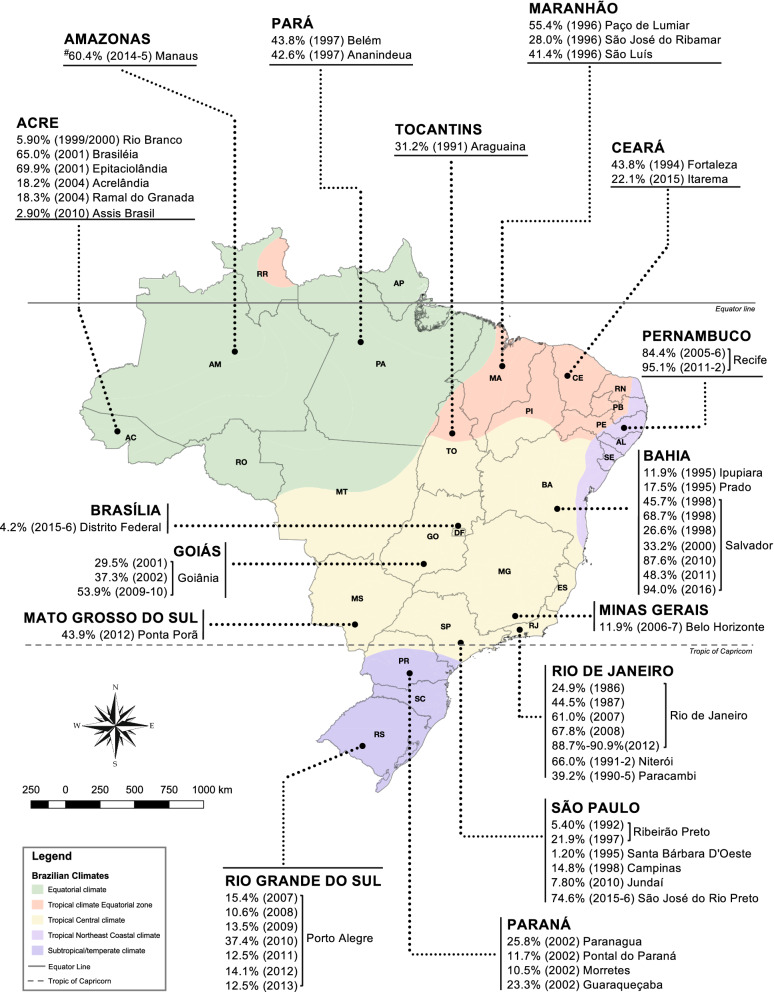
Summary of dengue prevalence studies reported between 1980 and 2020. QGIS software was used to plot tropical and subtropical areas along with study location, dengue prevalence and year of sample collection (Additional file 3: Table S2). All Brazilian states have confirmed DENV circulation but no prevalence data were available for some states (RO, RR, AP, PI, RN, PL, AL, SE, ES, DF and SC). *Manaus-AM 2015 data are from this study (refer Additional file 1: Table S1)

To understand overall antibody reactivity and distribution of DENV in Brazil, studies between 1980 and 2020 were identified from scientific databases and spatial analysis was performed (Figs. 5 and 6 and Additional file 3: Table S3 and Additional file 4: Table S4). Most of these studies used a different sampling strategy, age groups and dengue detection assay, which impede comparison between them (Additional file 1: Table S1). However, several large cities demonstrated an increase in DENV prevalence since first detection. Most Brazilian cities have more than one dengue serotype circulating, for example, in Manaus in Amazonas state all four DENV serotypes have been detected. Hence, this prevalence data may be important for future DENV vaccination and arbovirus disease control strategies.

In summary, we observed that a major part of our tested population had antibodies to DENV; however, the absence or low reactivity against several arboviruses makes this population susceptible for endemic and emerging arboviral diseases.

## Discussion

Emerging and re-emerging arbovirus infections pose a serious public health threat in tropical and subtropical regions worldwide [2, 3]. In this study, we observed that half of the study population was seropositive for dengue virus; however, over 90% of our study population was susceptible to endemic and emerging viruses in the absence of detectable antibodies. The presence of a large and susceptible population, as well as the abundant presence of mosquito vector populations, is an important factor that contributed to the recent large outbreaks of ZIKV and CHIKV reported in Brazil and other Latin American countries, upon the introduction of the viral diseases in the continent. Also, a lack of adequate molecular diagnostic tools, and the antibody cross-reactivity between these endemic viruses, contributed to their spread and delayed diagnosis.

All four dengue serotypes are endemic in Brazil, and at least two of the four serotypes circulate in most of the Brazilian states; refer to Additional file 4: Table S4 [7, 9, 26, 27]. For example, in Manaus, Amazonas state, all four DENV serotypes have been detected [28]. Likewise, prevalence has been shown to be in the range of 15 to 80%, depending on the population and region; in addition, the prevalence of DENV infection has been shown to increase with age [5, 7, 9, 19, 29]. We observed a maximum positivity of 58.4% for DENV-2 among our study population. Also, HIA titers for DENV-1 and -2 antibodies were higher compared to DENV-3 and 4, which may be partially explained by the late introduction of these viruses in Brazil [7, 26]. However, we did not observe any correlation between age and prevalence frequencies as observed in other studies. Military personnel are dynamic populations, and they move constantly from one region to another for duty; this factor could explain the discrepancy observed between the lack of correlation between age and prevalence in our study population as compared with the general population that resides in the same location over a period of time. The evaluated information suggests that an important factor in the development of the overwhelming epidemic of ZIKV and CHIKV in Brazil could be attributed to the low level or absence of pre-existing antibody levels, combined with the ample presence of *Aedes aegypti* in the urban setting [11, 29–31].

More individuals had antibodies reactive against the yellow fever vaccine (YFV) strain 17D compared to the wild-type strain. Moreover, the decreasing reactivity toward wild-type strain may be an indicator of a gradual decrease of antibody titers over time after vaccination. YFV booster doses at 10 years after primary vaccination in travelers to endemic regions, endemic populations and high-risk individuals such as military personnel are necessary to heighten the 17DD-YF-specific immune response and to achieve efficient immunity [32].

The presence of heterologous neutralizing antibody titers is inversely correlated with the severity of patients with a second DENV infection [8, 33–35]. Additionally, over 30% of the individuals had antibodies against major endemic and emerging *Flaviviruses* tested in this study. We cannot rule out multiple flavivirus infections in the same individual. These findings, along with other reports, indicate that SLEV, WNV, ILHV and ROCV circulation in Brazil is largely unknown, and there may be epidemiological implications of the co-circulation of these arboviruses [16, 19]. Overall, in vivo or cohort studies are needed to ascertain the role of multiple *Flavivirus* infections in cross-protection or induction of a severe disease [8, 13, 15, 36]. Regarding their role in disease and protection, low avidity antibodies against DENV have been shown to participate in severe disease; also poorly neutralizing antibodies can participate in antibody-dependent enhancement (ADE) in DENV infections [37–39]. Recent studies have demonstrated a role of DENV antibodies in causing ADE during ZIKV infection [40, 41]. On the other hand, several studies have also demonstrated a lack of ADE like cytokine storm and partial protective role of these flavivirus cross-reactive antibodies upon ZIKV infection [42, 43]. Nevertheless, comprehensive in vivo studies are necessary to ascertain the role of these cross-reactive antibodies in ADE during ZIKV and other *Flavivirus* infections.

MAYV is endemic in the Amazon region, and there have been imported cases in other regions of Brazil [21, 44]. A prevalence of more than 40% for MAYV has been described in some Amazonian communities [21]. However, we did not observe any positive samples for the *Alphavirus* tested in our study. Similarly, we observed very low prevalence of OROV belonging to *Peribunyaviridae*. OROV is still localized in the Amazon region and is responsible for causing neurological disease in urban and rural areas [45].

One of the limitations of the present study is that it was not feasible to perform a neutralization assay to distinguish between dengue serotypes and confirm or rule out multiple infections or cross-reactivity. HIA detects total reactive antibodies against the test antigen; previous studies have demonstrated a lower sensitivity of the HIA compared to ELISA. However, since no sensitivity and specificity values for each virus are available, we could not perform a statistical adjustment to estimate the true prevalence; here, we describe only the crude percentage reactivity for each virus tested in this study. Although most study participants were young male adults, these results are in accordance with previous studies on DENV prevalence [9, 29]. Furthermore, given the size and geographical differences in Brazil, the estimates from one region or state cannot be used to understand epidemiology from the whole of Brazil. On the other hand, soldiers are a high-risk group because of the activities they are involved in and their contact with endemic regions such as the Amazon rainforest. A very small proportion of the study population reported previous malaria infection, which suggests that most of the individuals lived in urban regions or have spent little time in the rural Amazon region, where 99.9% of the malaria cases are described [46]. This might explain the low level or absence of antibodies against arboviruses described in the Amazonian region, such as OROPV and MAYV [22, 47]. Moreover, only 17% of the individuals tested in this study self-reported dengue infection, which was three times lower than that observed by serology. These unreported dengue infections could be asymptomatic subclinical infections or self-limiting fever without diagnosis or clinically not diagnosed as dengue. Our cluster analysis suggests that ~ 60% of our study population comprised a low *Flavivirus*-positive population and was therefore susceptible to infection. Theoretically, susceptible military personnel could act as disease-spreading agents when returning to civil life when the combination of a susceptible population and specific vectors is present. More detailed serological surveys together with vector population assessment and viral detection strategies are needed to further characterize the extent of favorable factors that can contribute to future outbreaks and to forecast potential public health needs.

Overall, mosquito control measures and integrated vector management are essential for control of all arboviruses and were effective in controlling ZIKV and CHIKV outbreaks in Brazil and worldwide [48–50]. However, vector control precedes the decrease in herd immunity and the increase in the availability of susceptible populations [11, 33]. Adaptions of these emerging viruses to urban vectors like *Aedes aegypti* or *Culex quinquefasciatus* and the decreasing herd immunity to them might facilitate further epidemics of endemic and emerging arboviruses [2, 3]. Currently, differential clinical diagnosis is a major challenge when multiple viruses that cause similar clinical symptoms co-circulate [13, 14]; moreover, the lack of adequate diagnostic tools can limit early identification and efforts to block outbreaks [11, 51]. Warmer weather conditions brought on by the El Niño phenomenon and the destruction of the Amazon native forest can encourage faster breeding and maturation cycles for *Aedes* and *Anopheles* mosquito populations [52].

## Conclusions

A high percentage of individuals lacked antibodies against major endemic and emerging arboviruses, which makes them susceptible to further infections. Hence, improved vector and febrile syndrome surveillance to identify emerging pathogens is essential to prevent future outbreaks.

## Supplementary Information

**Additional file 1: Table S1.** Socio-demographic features of the study population.

**Additional file 2: Table S2.** Prevalence of antibodies against principal human arboviruses.

**Additional file 3: Table S3.** Dengue seroprevalence in Brazil reported between 1980 and 2020.

**Additional file 4: Table S4.** Distribution of dengue virus serotypes in Brazilian states.

**Additional file 5: Figure S1.** Distribution of dengue, Zika and Chikungunya virus cases in Brazil between 2014 and 2018. Hot spot detection maps were plotted using publicly available data for dengue (**a** and **b**), Zika (**c** and **d**) and Chikungunya (**e** and **f**) virus incidences between 2014 and 2018 (Ministry of Health Brazil, https://www.saude.gov.br/boletins-epidemiologicos). Please note the differences in the incidence rate scales for each virus. North region: Acre: AC, Amapá: AP, Amazonas: AM, Pará: PA, Rondônia: RO, Roraima: RR, Tocantins: TO; Northeast region: Alagoas: AL, Bahia: BA, Ceará: CE, Maranhão: MA, Paraíba: PB, Pernambuco: PE, Piauí: PI, Rio Grande do Norte: RN, Sergipe: SE; Midwest region: Goiás: GO, Mato Grosso: MT, Mato Grosso do Sul: MS, Distrito Federal (Federal District): DF; Southeast region: Espírito Santo: ES, Minas Gerais: MG, Rio de Janeiro: RJ, São Paulo: SP; South region: Paraná: PR, Rio Grande do Sul: RS, Santa Catarina: SC.

## Data Availability

The dataset supporting the conclusions of this article is included within the main manuscript and supplementary material.

## Abbreviations

HIA: Hemagglutination inhibition assay
YFV: Yellow fever virus
DENV: Dengue virus
ZIKV: Zika virus
SLEV: Saint Louis encephalitis virus
WNV: West Nile virus
ILHV: Ilheus virus
ROCV: Rocio virus
EEEV: Eastern equine encephalitis virus
WEEV: Western equine encephalomyelitis virus
MAYV: Mayaro virus
MUCV: Mucambo virus
CHIKV: Chikungunya virus
OROV: Oropouche virus
TCMV: Tacaiuma virus
CATUV: Catu virus
