# Supplementary material for: Prevalence of arbovirus antibodies in young healthy adult population in Brazil
Source: Parasit Vectors. 2021 Aug 14;14:403. doi: 10.1186/s13071-021-04901-4 (PMC8363865; doi:10.1186/s13071-021-04901-4)
Supplement: Supplementary file 1 — Additional file 1: Table S1. Socio-demographic features of the study population. [file 13071_2021_4901_MOESM1_ESM.docx]

**Additional file 1: Table S1. Socio-demographic features of the study population.**

| **Characteristic** | | **Value %** |
| --- | --- | --- |
| Age (years) | 20-29 | 71.0 |
|  | 30-39 | 22.0 |
|  | 40-49 | 7.1 |
| Race | Brown | 45.2 |
|  | Black | 9.5 |
|  | White | 35.0 |
|  | Yellow | 1.7 |
|  | Mixed | 8.5 |
| Family income | Minimum wage | 1.0 |
|  | Two minimum wages | 3.5 |
|  | Three minimum wages | 9.7 |
|  | Four minimum wages | 20.41 |
|  | Five minimum wages or more | 65.4 |
| Education level | Elementary school | 0.3 |
|  | High school | 54.0 |
|  | Graduate | 45.6 |
| Dengue infection ^1^ | Yes | 17.9 |
| Malaria infection ^1^ | Yes | 5.0 |
| Use Sistema Único de Saúde (SUS) ^2^ | Yes | 14.3 |

^1^Self-reported, ^2^Brazilian public funded health care system
