# Supplementary material for: Prevalence of arbovirus antibodies in young healthy adult population in Brazil
Source: Parasit Vectors. 2021 Aug 14;14:403. doi: 10.1186/s13071-021-04901-4 (PMC8363865; doi:10.1186/s13071-021-04901-4)
Supplement: Supplementary file 2 — Additional file 2: Table S2. Prevalence of antibodies against principal human arboviruses. [file 13071_2021_4901_MOESM2_ESM.docx]

**Additional file 2: Table S2.** Prevalence of antibodies against principal human arboviruses.

| Classification | Virus ^a^ | Total % ^b^ | Age 18-25 years % ^b^ | Age 26-35 years % ^b^ | Age >35 years % ^b^ |
| --- | --- | --- | --- | --- | --- |
| *Flaviviridae* |  |  |  |  |  |
| Yellow fever virus group | YFV/17D | 82.9 (247/298) | 82.5 (113/137) | 81.5 (97/119) | 87.8 (36/41) |
|  | YFV | 45.6 (136/298) | 42.3 (58/137) | 50.4 (60/119) | 41.5 (17/41) |
| Dengue virus group | DENV-1 | 56.7 (169/298) | 51.8 (71/137) | 62.2 (74/119) | 56.1 (23/41) |
|  | DENV-2 | 58.4 (174/298) | 53.3 (73/137) | 63.9 (76/119) | 58.5 (24/41) |
|  | DENV-3 | 56.4 (168/298) | 50.4 (69/137) | 61.3 (73/119) | 61.0 (25/41) |
|  | DENV-4 | 48.3 (144/298) | 43.1 (59/137) | 52.1 (62/119) | 53.7 (22/41) |
| Spondweni group | ZIKV | 30.9 (92/298) | 27.7 (38/137) | 31.9 (38/119) | 36.6 (15/41) |
| Japanese encephalitis virus group | WNV | 46.6 (139/298) | 40.9 (56/137) | 53.8 (64/119) | 43.9 (18/41) |
|  | SLEV | 54.7 (163/298) | 48.9 (67/137) | 63.0 (75/119) | 48.8 (20/41) |
| Ntaya virus group | ILHV | 51.3 (153/298) | 48.1 (66/137) | 54.6 (65/119) | 51.2 (21/41) |
|  | ROCV | 48.7 (145/298) | 43.8 (60/137) | 52.9 (63/119) | 51.2 (21/41) |
| *Peribunyaviridae* |  |  |  |  |  |
| Simbu serogroup | OROV | 0.7 (2/298) | 0.7 (1/137) | 0.8 (1/119) | 0 (0/41) |
| Guama serogroup | CATUV | 0.3 (1/298) | 0 (0/137) | 0 (0/119) | 2.4 (1/41) |
| Anopheles A serogroup | TCMV | 0 (0/298) | 0 (0/137) | 0 (0/119) | 0 (0/41) |
| *Togaviridae* |  |  |  |  |  |
| Semliki forest virus complex | MAYV | 0 (0/298) | 0 (0/137) | 0 (0/119) | 0 (0/41) |
|  | CHIKV | 0 (0/298) | 0 (0/137) | 0 (0/119) | 0 (0/41) |
| Eastern Equine Encephalitis Complex | EEEV | 0 (0/298) | 0 (0/137) | 0 (0/119) | 0 (0/41) |
| Western Equine Encephalitis Complex | WEEV | 0 (0/298) | 0 (0/137) | 0 (0/119) | 0 (0/41) |
| Venezuelan Equine Encephalitis Complex | MUCV | 0 (0/298) | 0 (0/137) | 0 (0/119) | 0 (0/41) |
| Negatives |  | 15.8 (47/298) | 16.1 (22/137) | 16.8 (20/119) | 12.2 (5/41) |

^a^ *Flaviviridae* (*Flavivirus* genus): Yellow fever virus (YFV), Dengue virus (DENV) serotypes 1 to 4 (DENV-1, DENV-2, DENV-3 and DENV-4), Zika virus (ZIKV), Saint Louis Encephalitis virus (SLEV), West Nile virus (WNV), Ilheus virus (ILHV) and Rocio virus (ROCV); *Peribunyaviridae* (*Orthobunyavirus* genus): Oropouche virus (OROV), Catu virus (CATUV) and Tacaiuma virus (TCMV); *Togaviridae* (*Alphavirus* genus): Mayaro virus (MAYV), Chikungunya virus (CHIKV), Eastern Equine Encephalitis virus (EEEV), Western Equine Encephalomyelitis virus (WEEV) and Mucambo virus (MUCV).

^b^ HIA titre ≥ 1:20
