# Supplementary material for: Prevalence of arbovirus antibodies in young healthy adult population in Brazil
Source: Parasit Vectors. 2021 Aug 14;14:403. doi: 10.1186/s13071-021-04901-4 (PMC8363865; doi:10.1186/s13071-021-04901-4)
Supplement: Supplementary file 4 — Additional file 4: Table S4. Distribution of dengue virus serotypes in Brazilian states. [file 13071_2021_4901_MOESM4_ESM.docx]

**Additional file 4: Table S4.** Distribution of dengue virus serotypes in Brazilian states.

| Geographical regions and states | Serotypes confirmed^a,b^ |
| --- | --- |
| North Region |  |
| Acre (AC) | DENV-1 |
| Amazonas (AM)^c^ | DENV-1, DENV-2, DENV-3 and DENV-4 |
| Amapá (AP) | DENV-1 |
| Pará (PA) | DENV-1, DENV-2 and DENV-4 |
| Rondônia (RO) | DENV-1 and DENV-4 |
| Roraima (RR) | DENV-1, DENV-2, DENV-3 and DENV-4 |
| Tocantins (TO) | DENV-1, DENV-2, DENV-3 and DENV-4 |
| Northeast Region |  |
| Alagoas (AL) | DENV-1 and DENV-4 |
| Bahia (BA) | DENV-1 and DENV-4 |
| Ceará (CE) | DENV-1, DENV-2, DENV-3 and DENV-4 |
| Maranhão (MA)^d^ | DENV-1, DENV-2, DENV-3 and DENV-4 |
| Paraíba (PB) | DENV-1, DENV-2, DENV-3 and DENV-4 |
| Pernambuco (PE) | DENV-1, DENV-2, DENV-3 and DENV-4 |
| Piauí (PI) | DENV-1, DENV-2 and DENV-3 |
| Rio Grande do Norte (RN) | DENV-1, DENV-2 and DENV-4 |
| Sergipe (SE) | DENV-1 and DENV-4 |
| Midwest Region |  |
| Distrito Federal (DF) | DENV-1, DENV-2, DENV-3 and DENV-4 |
| Goiás (GO) | DENV-1, DENV-2, DENV-3 and DENV-4 |
| Mato Grosso (MT)^d,e^ | DENV-1 and DENV-4 |
| Mato Grosso do Sul (MS) | DENV-1, DENV-2 and DENV-4 |
| Southeast Region |  |
| Espírito Santo (ES) | DENV-1 and DENV-4 |
| Minas Gerais (MG) | DENV-1, DENV-2, DENV-3 and DENV-4 |
| Rio de Janeiro (RJ) | DENV-1, DENV-2^d^, DENV-3^f^ and DENV-4 |
| São Paulo (SP) | DENV-1, DENV-2, DENV-3 and DENV-4 |
| South Region |  |
| Paraná (PR) | DENV-1, DENV-2 and DENV-4 |
| Rio Grande do Sul (RS) | DENV-1, DENV-2^e^, DENV-3^e^ and DENV-4 |
| Santa Catarina (SC) | DENV-1 |

DENV serotypes reported in Brazil by the Ministry of Health. Data are organized by geographical regions and the states they include. ^a^Data reported by Brazilian Ministry of Health until the epidemiological week 16-31/12/17 to 21/04/18. Source: http://portalsaude.saude.gov.br. ^b^Data obtained in Fares et al. (2015) [6]. ^c^Data obtained in Bastos et al. (2012) [28]. ^d^DENV serotype (s) found only in year 2015. ^e^DENV serotype (s) found only in year 2016. ^f^DENV serotype (s) found only in year 2017.
