# Supplementary figures and images for: Prevalence of arbovirus antibodies in young healthy adult population in Brazil
Source: Parasit Vectors. 2021 Aug 14;14:403. doi: 10.1186/s13071-021-04901-4 (PMC8363865; doi:10.1186/s13071-021-04901-4)

a

Dengue

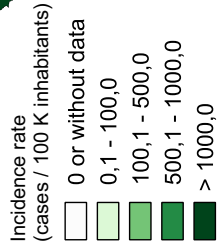

2014 2015 2016 2017 2018

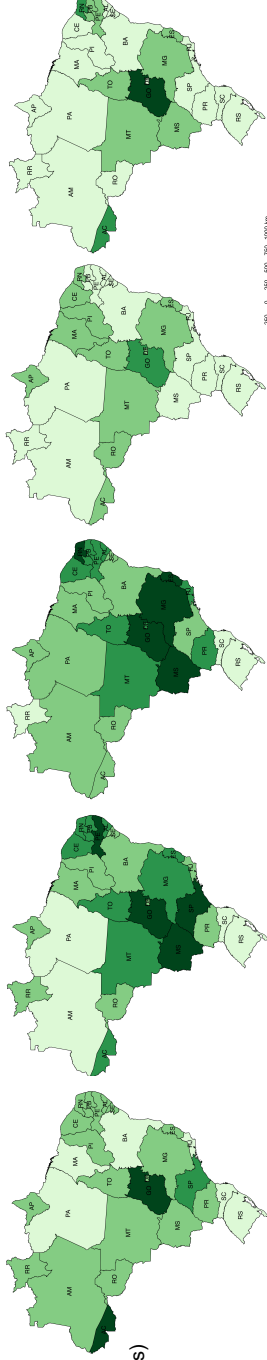

b

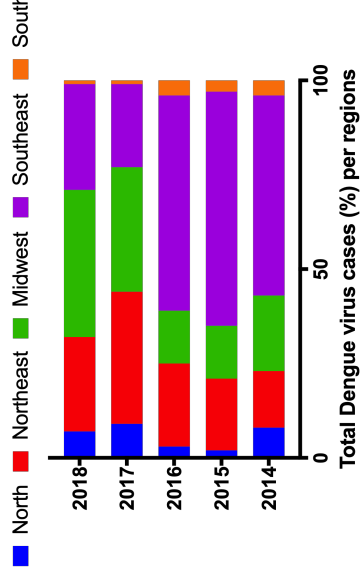

c

Zika

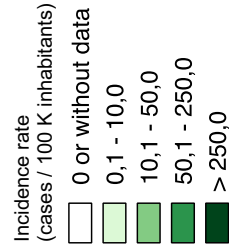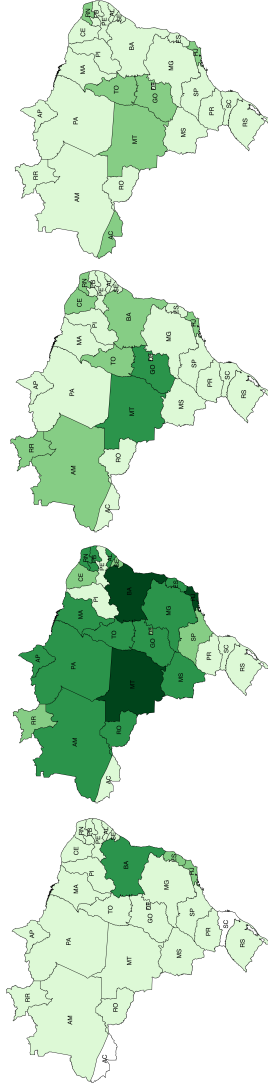

d

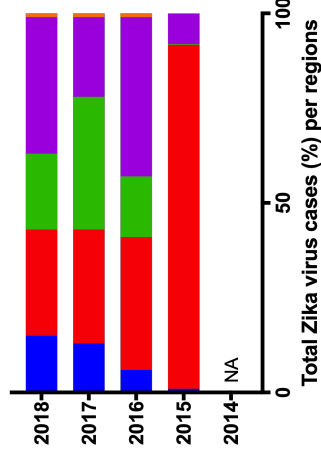

e

Chikungunya

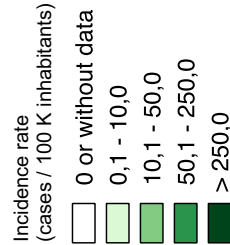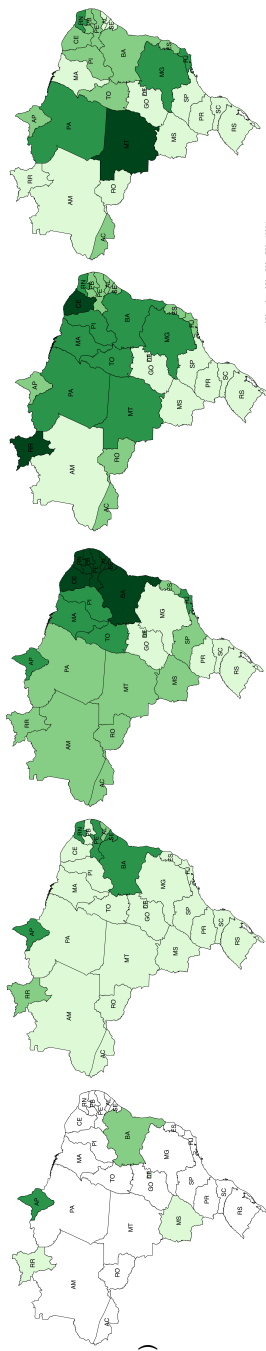

f

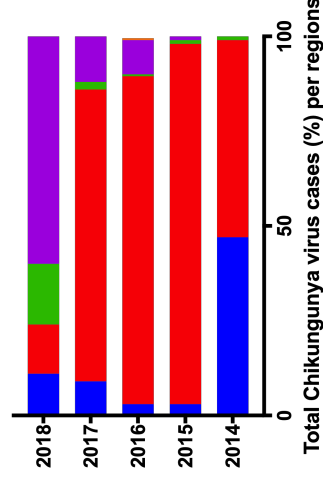

Supplement: Supplementary file 5 — Additional file 5: Figure S1. Distribution of dengue, Zika and Chikungunya virus cases in Brazil between 2014 and 2018. Hot spot detection maps were plotted using publicly available data for dengue (a and b), Zika (c and d) and Chikungunya (e and f) virus incidences between 2014 and 2018 (Ministry of Health Brazil, https://www.saude.gov.br/boletins-epidemiologicos). Please note the differences in the incidence rate scales for each virus. North region: Acre: AC, Amapá: AP, Amazonas: AM, Pará: PA, Rondônia: RO, Roraima: RR, Tocantins: TO; Northeast region: Alagoas: AL, Bahia: BA, Ceará: CE, Maranhão: MA, Paraíba: PB, Pernambuco: PE, Piauí: PI, Rio Grande do Norte: RN, Sergipe: SE; Midwest region: Goiás: GO, Mato Grosso: MT, Mato Grosso do Sul: MS, Distrito Federal (Federal District): DF; Southeast region: Espírito Santo: ES, Minas Gerais: MG, Rio de Janeiro: RJ, São Paulo: SP; South region: Paraná: PR, Rio Grande do Sul: RS, Santa Catarina: SC. [file 13071_2021_4901_MOESM5_ESM.pdf]
